# Narirutin mitigates neuroinflammation following traumatic brain injury and modulates microglial polarization via the JAK2/STAT3 signaling pathway

**DOI:** 10.3389/fneur.2025.1680790

**Published:** 2025-10-22

**Authors:** Hebo Zhang, Junming Han, Chaolong Yan, Jiannan Mao, Huiying Yan, Wei Jin

**Affiliations:** Department of Neurosurgery, Nanjing Drum Tower Hospital, Affiliated Hospital of Medical School, Nanjing University, Nanjing, China

**Keywords:** Narirutin, traumatic brain injury, microglia, neuroinflammation, JAK2/STAT3 pathway

## Abstract

**Background:**

Traumatic brain injury (TBI) causes irreversible cerebral damage characterized by neuroinflammation and neuronal injury, representing a critical factor contributing to poor prognosis in TBI patients. While existing studies have demonstrated Narirutin’s (Nar) neuroprotective effects in Parkinson’s disease, research on Nar in the context of traumatic brain injury remains markedly limited. This investigation elucidates Nar’s protective mechanisms in murine TBI models.

**Methods:**

The behavioral tests of TBI model mice were conducted using the Morris water maze method. HE staining, Nissl staining and immunohistochemistry were performed. Western blotting was used to detect inflammatory indicators, and immunofluorescence was used to detect microglial polarization.

**Results:**

Nar significantly reduced the inflammatory response and abnormal activation of signaling pathways induced by TBI. This effect is achieved by reducing the expression levels of NLRP3, IL-1β, and IL-6, promoting M2 polarization in microglia, and inhibiting the phosphorylation of JAK and STAT within the TBI model.

**Conclusion:**

In conclusion, our research results indicate that Nar can improve neuroinflammation in TBI model mice, demonstrating excellent anti-inflammatory effects and neuroprotective properties, providing potential therapeutic strategies for the clinical treatment of TBI.

## 1 Introduction

Traumatic brain injury (TBI) is a severe neurological disorder affecting 50 million people globally each year, frequently causing patient mortality and disability while imposing a substantial worldwide health burden (1). In China, due to its large population, TBI incidence exceeds that of other countries, with an estimated mortality rate of approximately 13 per 100,000 (2). TBI results from mechanical impact to the brain, where primary injuries directly damage neurons, blood vessels, and the blood–brain barrier (BBB) (3–5). Secondary injuries encompass mitochondrial dysfunction, inflammatory factor release, oxidative stress formation, and other processes that further induce neuronal apoptosis and necrosis (6–8). Despite extensive clinical and fundamental research on TBI’s pathophysiological mechanisms by domestic and international scholars, the precise injury mechanisms remain undetermined, leading to a lack of effective clinical treatments (9, 10). Consequently, elucidating the underlying mechanisms of TBI is imperative.

In neuroinflammation triggered by Traumatic Brain Injury, microglia, the primary immune cells of the Central Nervous System (CNS), play a pivotal role in regulating the neuroinflammatory process (11, 12). Acting as key regulators of the CNS immune response, microglia rapidly transition to an activated state following injury, a phenomenon collectively termed microglial polarization (13, 14). Activated microglia polarize into two distinct types, M1 and M2, which exhibit significantly different functionality and morphology (15, 16). M1-type cells primarily exert bactericidal and pro-inflammatory effects, whereas M2-type cells are characterized by anti-inflammatory actions and functions that promote neural repair (17–19). In recent years, researchers have found that JAK/STAT signaling pathway can be activated after TBI and participate in microglia polarity-related neuroinflammation (20). More and more neurological studies have shown that pharmacological intervention to inhibit JAK/STAT pathway to reduce neuroinflammation and thereby protect neurons is effective (21).

Narirutin (Nar) is a flavonoid commonly found in citrus fruits such as grapefruits, oranges, and tangerines (22, 23). It exhibits blood–brain barrier permeability and diverse biological activities, including antioxidant, anti-inflammatory, and neuroprotective properties (24–26). Notably, a recent study demonstrated that Nar’s anti-inflammatory effects alleviate inflammatory responses by inhibiting the activation of the NOD-like receptor protein 3 (NLRP3) inflammasome in macrophages (27). This anti-inflammatory action has been validated in a rat model of transient cerebral ischemia–reperfusion injury (28). Nar also exerts anti-inflammatory effects via the PI3K/AKT and JAK2/STAT3 signaling pathways (29, 30). It significantly reduces expression levels of IL-1β, TNF-*α*, and IL-6, thereby ameliorating the inflammatory microenvironment (31). Furthermore, Nar mitigates dopaminergic neuronal loss and improves motor dysfunction in Parkinson’s disease (PD) mouse models (32, 33). However, research on Nar in the context of traumatic brain injury remains notably unexplored.

In this study, we investigated the underlying molecular mechanisms by which Nar alleviates neuroinflammation following traumatic brain injury, with a specific focus on its anti-inflammatory effects. The work aims to provide a potential therapeutic strategy for mitigating secondary brain injury after TBI.

## 2 Materials and methods

### 2.1 Experimental animals

Male C57BL/6 mice aged 8–9 weeks (body weight: 18–22 g) were housed under controlled temperature and humidity conditions with a 12-h light/dark cycle. All animals were handled and treated in accordance with ethical standards, provided ad libitum access to food and water.

### 2.2 *In vivo* model of TBI

Mice were randomly divided into four groups: sham, TBI, Nar (50 mg/kg) and vehicle. The mouse TBI model was established using controlled cortical impact (CCI) (34). Following anesthesia via intraperitoneal injection of 3% pentobarbital sodium (60 mg/kg), mice were secured in a stereotaxic frame in the prone position. A craniotomy with a diameter of 4 mm was conducted on the left cerebral hemisphere, which was precisely positioned 3 mm lateral to the midline and 3 mm posterior to bregma. A moderate TBI model was produced with craniocerebral strike apparatus using a 3 mm diameter impactor (velocity 1.5 m/s; duration 50 ms; depth 1 mm), as previously described. Mice were then transferred to heating pads for postoperative recovery. For the sham-operated group: Scalp incision and craniotomy were performed without cortical impact. The scalp was subsequently sutured, and mice were placed on heating pads for natural awakening. One hour after TBI, Nar mice were given intragastric Nar once daily for three days, and vehicle mice were given saline by gavage. Nar was obtained from Shanghai Pure One Biotechnology (purity >98%; Shanghai, China) and dissolved in saline.

### 2.3 Modified mouse neurological function score (mNSS score)

Neurological function was assessed using the modified Neurological Severity Score (mNSS) following traumatic brain injury (TBI). This 18-point scale demonstrates an inverse correlation between numerical values and functional capacity, where elevated scores correspond to greater neurological impairment. Specifically: a score of 0 indicates intact neurological function; scores of 1–6 represent mild impairment; scores of 7–12 denote moderate impairment; and scores of 13–18 signify severe neurological deficits.

### 2.4 Nissl staining

Intracardiac perfusion was removed from intact brain tissues, fixed in 4% paraformaldehyde for 24 h, then dehydrated with a gradient of 15 and 30% sucrose. Sections were, frozen and sectioned at 20 μm, and then stained with Nissen stain according to the manufacturer’s instructions (BL1039A, Biosharp, Hefei, China). Sections were photographed under a Leica Thunder microscope, and the number of positive cells in the brain sections was counted.

### 2.5 Morris water maze (MWM)

The Morris Water Maze (MWM) assessed spatial learning and memory acquisition. Over four consecutive days, mice completed four daily trials locating a hidden platform within an opaque pool. A 120-s probe trial followed on day five with the platform removed, quantifying navigation patterns through escape latency, swimming velocity, and target quadrant occupancy time.

### 2.6 Western blot

Hippocampal tissues were homogenized in RIPA lysis buffer containing protease inhibitors, followed by centrifugation to collect supernatants. Protein concentrations were determined using the BCA assay. Protein samples were separated by SDS-PAGE electrophoresis and transferred onto PVDF membranes. After blocking with 5% skim milk, membranes were incubated with primary antibodies at 4 °C overnight, followed by 1-h incubation with enzyme-conjugated secondary antibodies at room temperature. Protein bands were visualized using ECL reagents and quantified objectively with ImageJ software.

### 2.7 Hematoxylin and eosin (HE) staining

To evaluate tissue morphology, paraffin-embedded brain sections underwent HE staining. Following deparaffinization in xylene, samples were rehydrated through a graded ethanol series. Nuclei were stained with hematoxylin (5 min), followed by cytoplasmic counterstaining with eosin (3 min). Sections were subsequently dehydrated, cleared, and examined under an Olympus BX53 microscope (Olympus Optical Co., Tokyo, Japan).

### 2.8 Immunofluorescence (IF)

Following fixation, mouse brains underwent cryoprotection in graded sucrose solutions (20% → 30%), embedding, and cryosectioning at 30 μm. Selected sections were sequentially processed through PBST washes, 1-h blocking in 3% BSA, and overnight incubation at 4 °C with primary antibodies (CD206/CD68; 1:300). After rigorous PBST rinses (3 × 10 min), sections were incubated for 1 h with species-matched secondary antibodies: Alexa Fluor 546 donkey anti-rabbit IgG or Alexa Fluor 488 donkey anti-goat IgG. Nuclei were counterstained with DAPI (10 min) prior to mounting, 4-h slide baking, and sealing with antifade medium. Fluorescence imaging was conducted using a Zeiss microscope followed by quantitative analysis.

### 2.9 Statistical analysis

All data are expressed as mean ± standard error of the mean. Following verification of normality and homogeneity of variance (chi-square tests), statistical analyses were conducted using Prism 9 (GraphPad Software, La Jolla, CA, USA) and SPSS 25.0 (SPSS Inc.), employing: t-tests for two-group comparisons and one-way ANOVA with Bonferroni *post hoc* tests for multi-group independent variables, with statistical significance defined as *p* < 0.05.

## 3 Results

### 3.1 Nar improves neurological impairment following traumatic brain injury

**Figure 1:**
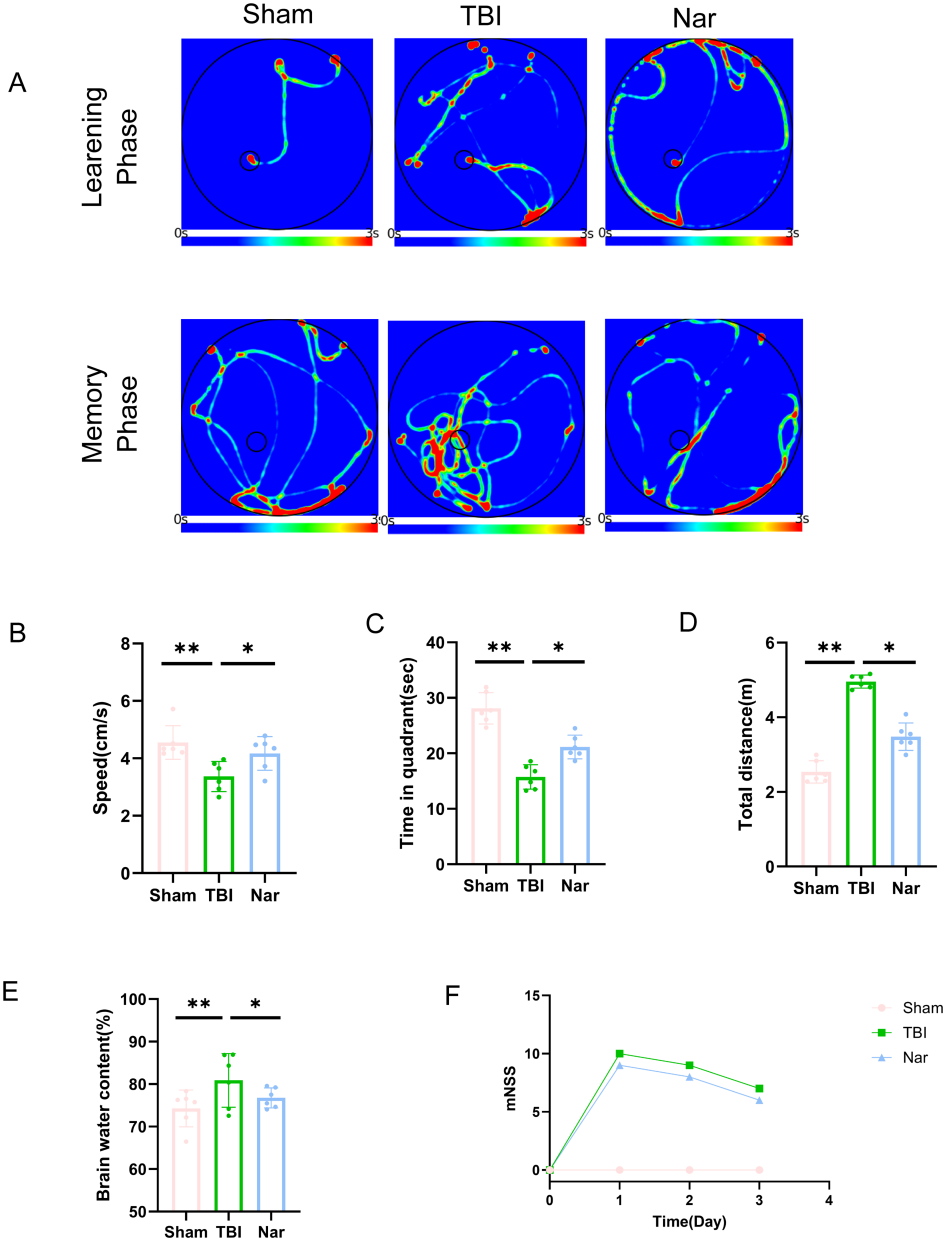
Nar ameliorates motor dysfunction following TBI. **(A)** Representative spatial learning and memory traces from the Morris water maze test. **(B)** Quantitative analysis of swimming velocity in the water maze. **(C)** Quantification of time spent in the target quadrant during water maze probe trials. **(D)** Quantitative assessment of total swimming distance in the water maze. **(E)** Cerebral water content quantification in mouse brains. **(F)** Modified Neurological Severity Scores (mNSS) of mice on days 1, 2, and 3 post-TBI induction. ns; *p* > 0.05; *p ≤ 0.05; **p ≤ 0.01; ***p ≤ 0.001; ****p ≤ 0.0001; *n* = 6.

MWM testing was employed to evaluate the neuroprotective effects of Nar. We investigated Nar’s potential protective role against TBI-induced learning and memory impairments (Figure 1A). Throughout the training phase, Nar-treated mice exhibited significantly shorter escape latencies to locate the hidden platform compared to the TBI group, indicating Nar-facilitated recovery of compromised learning ability post-TBI (Figure 1B). Furthermore, during the probe trial (platform-removed memory assessment phase), Nar-treated groups spent substantially more time in the target quadrant than TBI controls (Figures 1C,D). These results demonstrate Nar significantly mitigates TBI-induced deficits in both learning capacity and spatial memory retention. To obtain a more precise assessment of neurological functional recovery, we implemented the mNSS scale (Figure 1F). Quantitative analysis revealed markedly greater neurological improvement in Nar-treated mice versus the TBI group. We additionally evaluated cerebral edema—a critical manifestation of secondary brain injury—by calculating brain water content through wet-to-dry weight ratio measurement (Figure 1E). Results showed Nar treatment significantly reduced cerebral edema compared to TBI controls. Collectively, these findings indicate Nar attenuates secondary injury progression and accelerates functional recovery following TBI.

### 3.2 Nar alleviated TBI-induced nerve damage and inflammation

**Figure 2:**
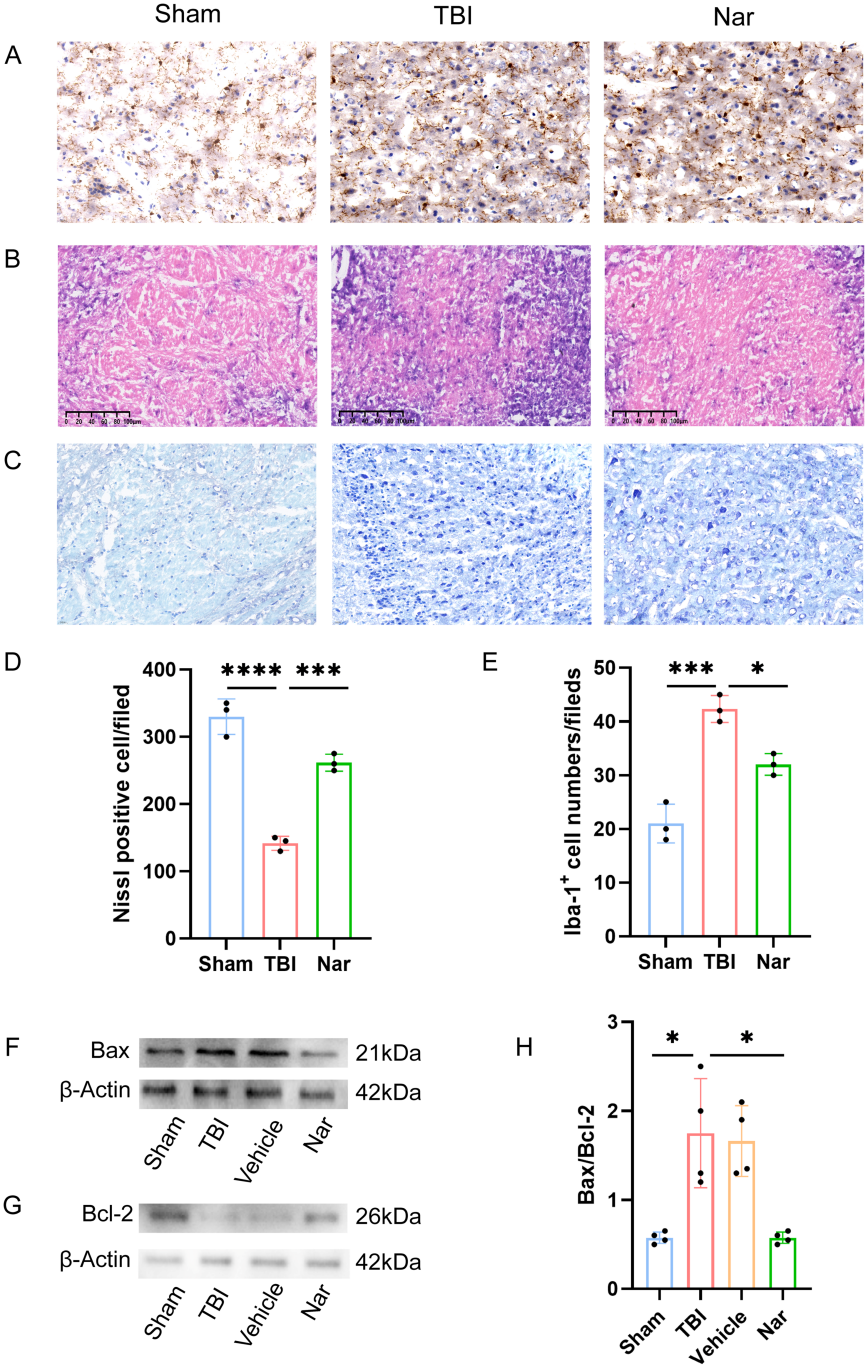
Nar attenuates microglial activation and inflammatory responses post-TBI in vivo. **(A)** Representative immunohistochemical images of Iba-1^+^ microglia in brain tissues from Sham, TBI, and Nar-treated mice. **(B)** H&E staining of mouse brain tissue sections. **(C)** Nissl-stained representative sections from Sham, TBI, and Nar-treated mouse brains. **(D)** Statistical analysis of Nissl-positive cell counts based on representative histological images. **(E)** Quantitative assessment of microglial density derived from immunohistochemical images. **(F)** Western blot analysis of Bax protein levels in mouse brain tissues. **(G)** Western blot analysis of Bcl-2 protein levels in mouse brain tissues. **(H)**. Statistical analysis of the pro-apoptotic (Bax) to anti-apoptotic (Bcl-2) protein ratio in mouse brain tissues. ns; *p* > 0.05; *p ≤ 0.05; **p ≤ 0.01; ***p ≤ 0.001; ****p ≤ 0.0001; **(A–E)** *n* = 3, **(F–H)** *n* = 4.

Histopathological analysis via H&E staining demonstrated that Nar treatment ameliorated neuronal damage in TBI mice. The TBI group exhibited characteristic neuronal pathologies including reduced soma volume, pyknotic nuclei, neuronal degeneration, and inflammatory cell infiltration. In contrast, Nar-treated animals showed significantly attenuated cerebral inflammation and cellular degeneration compared to TBI controls (Figure 2B). Immunohistochemical evaluation using the microglial marker Iba-1 revealed substantially increased Iba-1-positive cell counts and enhanced microglial activation in TBI mice (Figures 2A,E). Notably, Nar administration significantly reduced Iba-1-positive cell numbers relative to untreated TBI counterparts, indicating effective suppression of post-TBI microglial hyperactivation.

### 3.3 Nar ameliorates TBI-induced neuronal apoptosis

We evaluated the impact of Nar on TBI-induced neuronal apoptosis via Nissl staining (Figure 2C). Results demonstrated a significant reduction in neuronal density post-TBI, with injured neurons exhibiting cytoplasmic shrinkage and substantially diminished Nissl bodies. However, Nar administration significantly increased neuronal counts and markedly restored Nissl body integrity, indicating neuroprotection through mitigation of TBI-induced neuronal damage (Figure 2D). We evaluated protein expression of apoptosis-related markers Bax and Bcl-2 (Figures 2F,G). Western blot analysis revealed significantly increased Bax levels post-TBI, which were substantially reduced by Nar treatment. Conversely, Bcl-2 expression was significantly decreased after TBI but markedly restored following Nar administration. To comprehensively assess Nar’s impact on apoptosis, we calculated the Bax/Bcl-2 ratio - a critical indicator of apoptotic propensity (Figure 2H). Compared to controls, the TBI group exhibited a significantly higher ratio, indicating enhanced apoptosis. Nar treatment significantly lowered the Bax/Bcl-2 ratio, demonstrating effective attenuation of TBI-induced apoptotic activation. These integrated results establish that Nar mediates neuroprotection, likely through amelioration of TBI-triggered neuronal damage and restoration of apoptotic homeostasis.

### 3.4 Nar treatment suppresses microglia-mediated neuroinflammation following TBI

**Figure 3:**
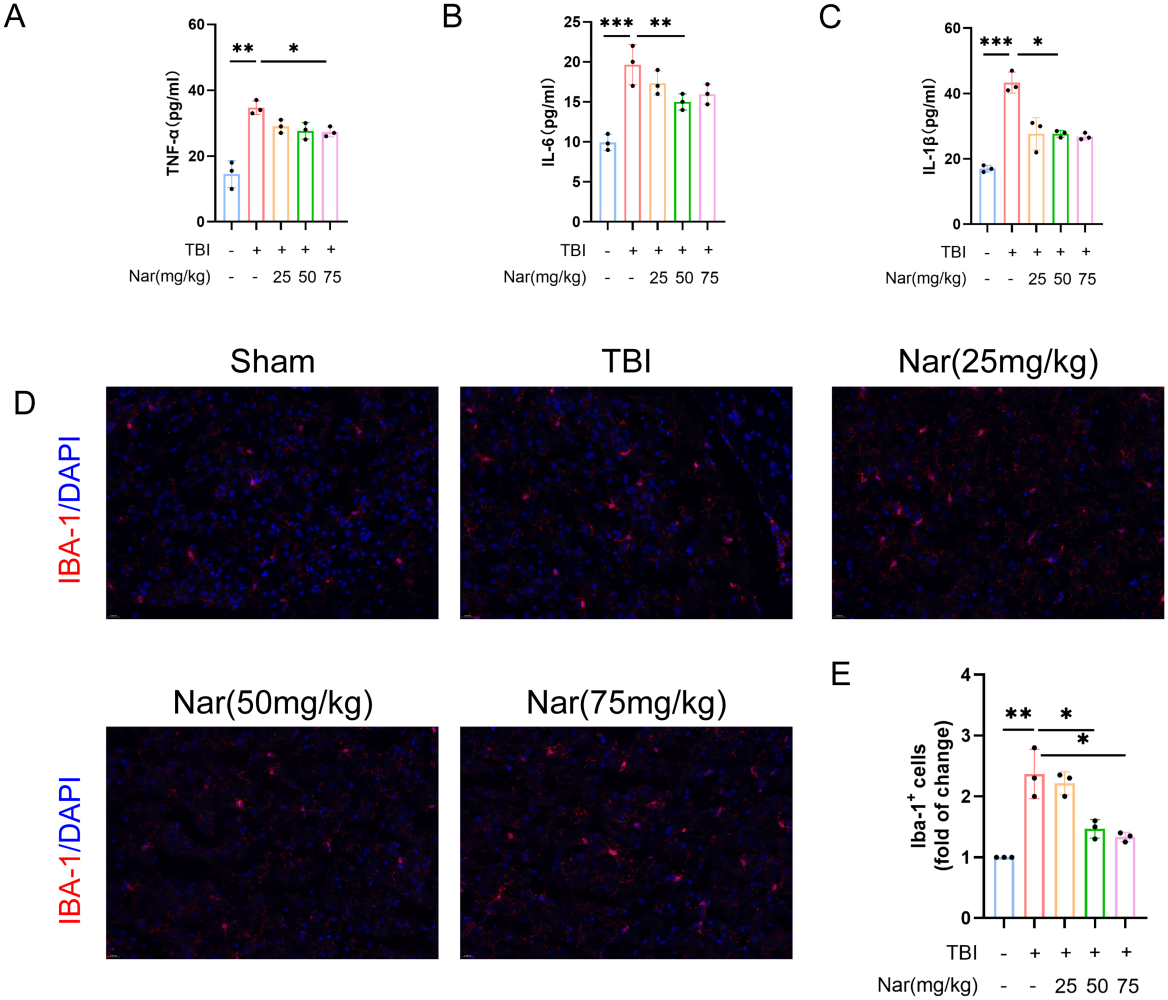
Nar treatment suppresses microglia-mediated inflammation post-TBI. **(A-C)** Serum levels of TNF-α **(A)**, IL-6 **(B)**, and IL-1β **(C)** in mice (*n* = 3 mice per group). **(D)** Tissue sections immunostained with the microglial marker IBA-1 antibody (red) and counterstained with DAPI for nuclei (blue). Scale bar = 200 μm. **(E)** Quantification of IBA-1-positive cells in brain tissues (*n* = 3 mice per group). ns; *p* > 0.05; *p ≤ 0.05; **p ≤ 0.01; ***p ≤ 0.001; ****p ≤ 0.0001; *n* = 3.

**Figure 4:**
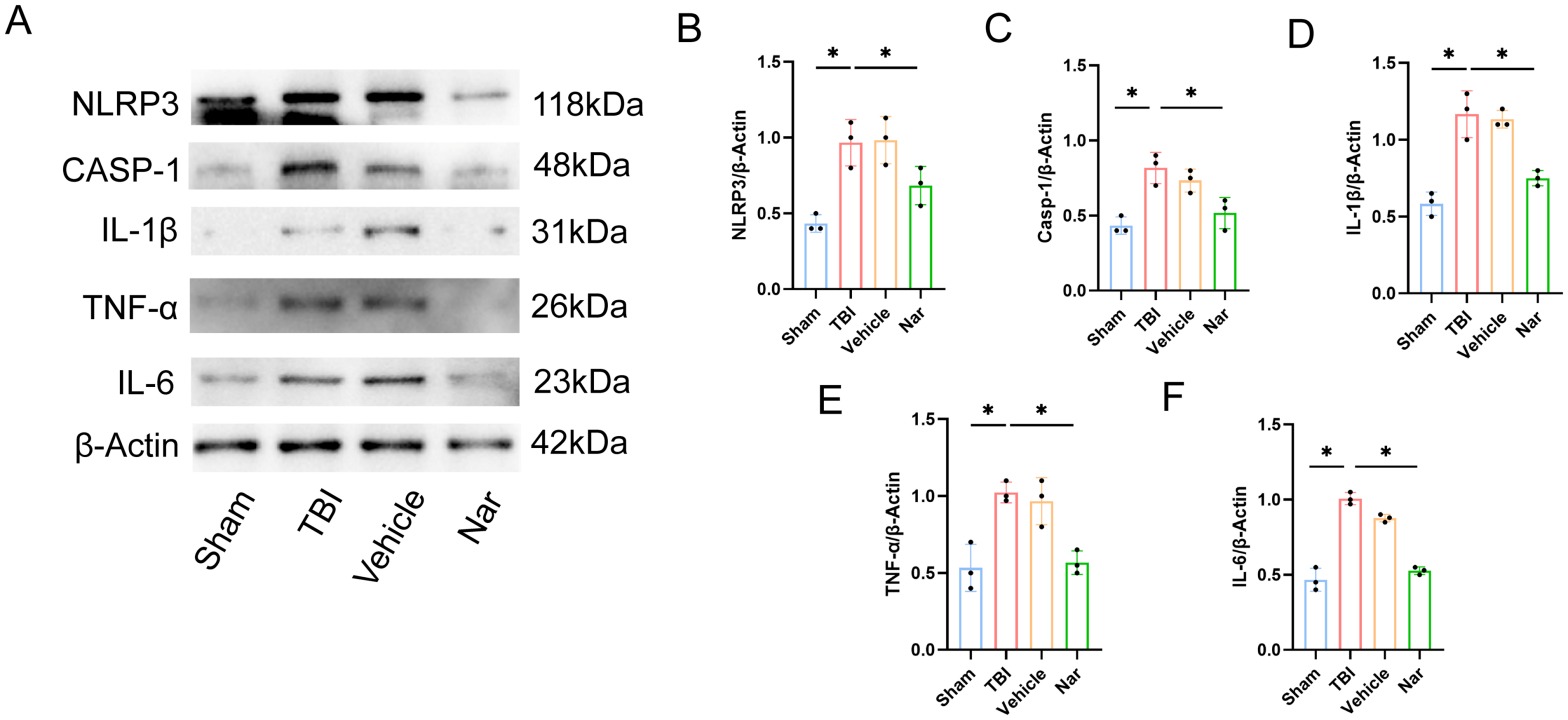
Nar suppresses inflammatory responses post-TBI. **(A–F)** Representative Western blot bands and semi-quantitative analysis of protein expression for NLRP3, CASP-1, IL-1β, TNF-α, and IL-6. ns; *p* > 0.05; *p ≤ 0.05; **p ≤ 0.01; ***p ≤ 0.001; ****p ≤ 0.0001; *n* = 3.

We measured the effects of Nar on serum levels of pro-inflammatory cytokines. ELISA results demonstrated that TBI significantly elevated serum concentrations of TNF-α, IL-1β, and IL-6, while Nar administration effectively counteracted these changes (Figures 3A–C). Given that neuroinflammation is primarily mediated by microglia, we further evaluated Nar’s impact on microglial activation using immunofluorescence staining. Notably, the TBI group exhibited both a significant increase in Iba-1-positive microglial numbers and cellular hypertrophy—morphological indicators of microglial activation (Figures 3D,E). Nar treatment reduced activated microglial counts in a dose-dependent manner. Collectively, these data indicate that Nar suppresses microglial activation and inhibits systemic production of pro-inflammatory cytokines. Western blot analysis of NLRP3, IL-1β, Casp-1, TNF-*α*, and IL-6 revealed significant upregulation of these proteins in the TBI group (Figures 4A–F). Nar treatment effectively downregulated their expression levels. Collectively, these results demonstrate that Nar attenuates neuroinflammation.

### 3.5 Nar promotes M1-to-M2 microglial transition post-TBI

**Figure 5:**
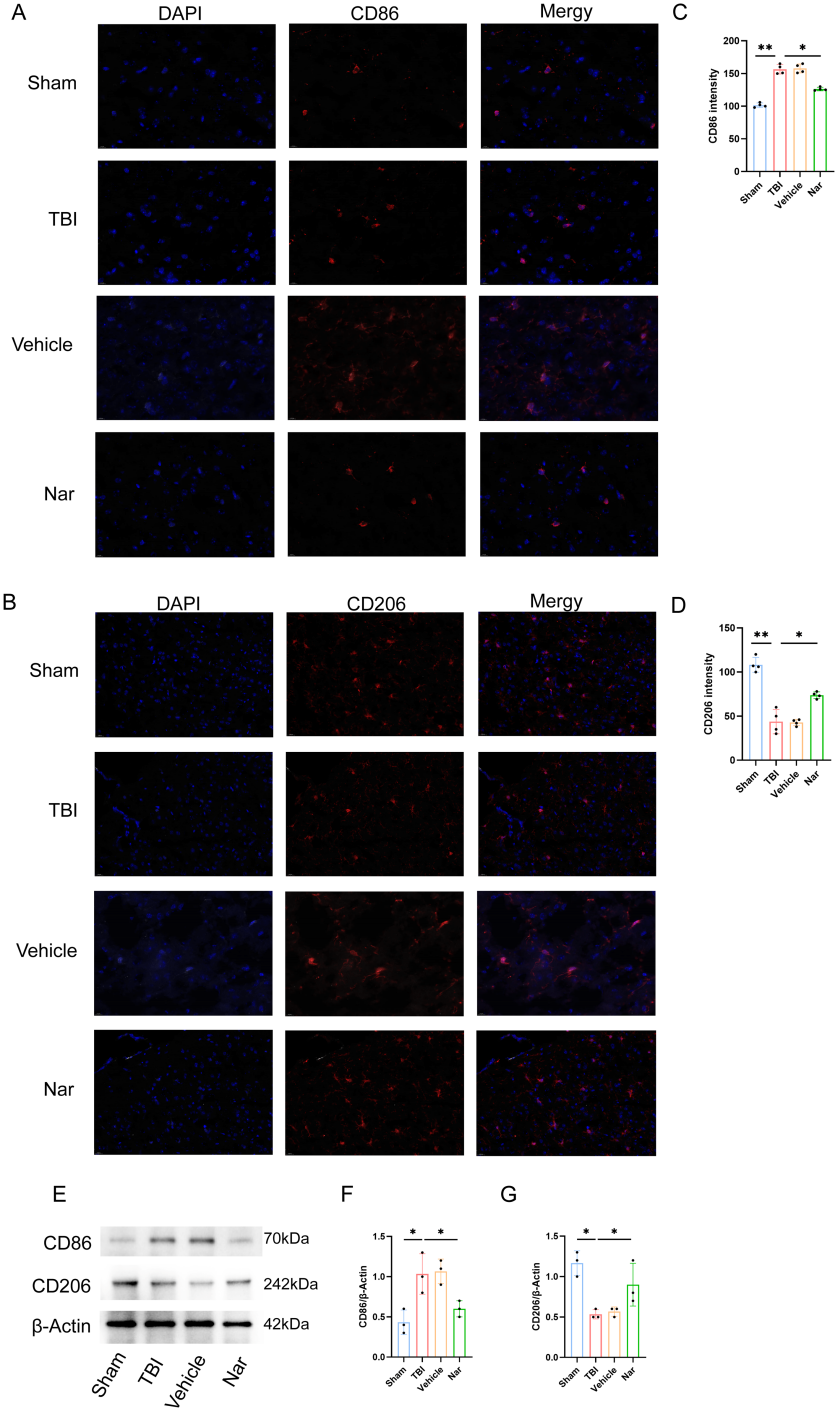
Nar drives microglial polarization toward anti-inflammatory phenotype after TBI. **(A,B)** CD86 (M1, red) and CD206 (M2, red) immunofluorescence with DAPI (blue) in brain sections. **(C,D)** CD86^+^ cell counts. **(E–G)** CD206^+^ cell quantification. **(H–J)** Representative blots and densitometry for CD86/CD206 proteins. ns; *p* > 0.05; *p ≤ 0.05; **p ≤ 0.01; ***p ≤ 0.001; ****p ≤ 0.0001; *n* = 3.

To determine Nar’s effects on microglial polarization post-TBI, we identified M1 and M2 phenotypes using immunofluorescence staining for CD68 and CD206, respectively (Figures 5A–D). Compared to controls, the TBI group exhibited significantly increased CD68^+^ cells in the hippocampus. In contrast, Nar treatment substantially reduced this pro-inflammatory microglial population relative to TBI counterparts. Moreover, Nar significantly increased CD206^+^ microglia compared to the TBI group. Western blot analysis further confirmed that Nar treatment significantly suppressed CD86 expression while promoting CD206 expression (Figures 5E–G). Collectively, these findings demonstrate that Nar facilitates a phenotypic shift from pro-inflammatory M1 to anti-inflammatory M2 polarization in microglia following TBI, thereby exerting neuroprotective effects.

### 3.6 Nar exerts its effects through the JAK2/STAT3 pathway

**Figure 6:**
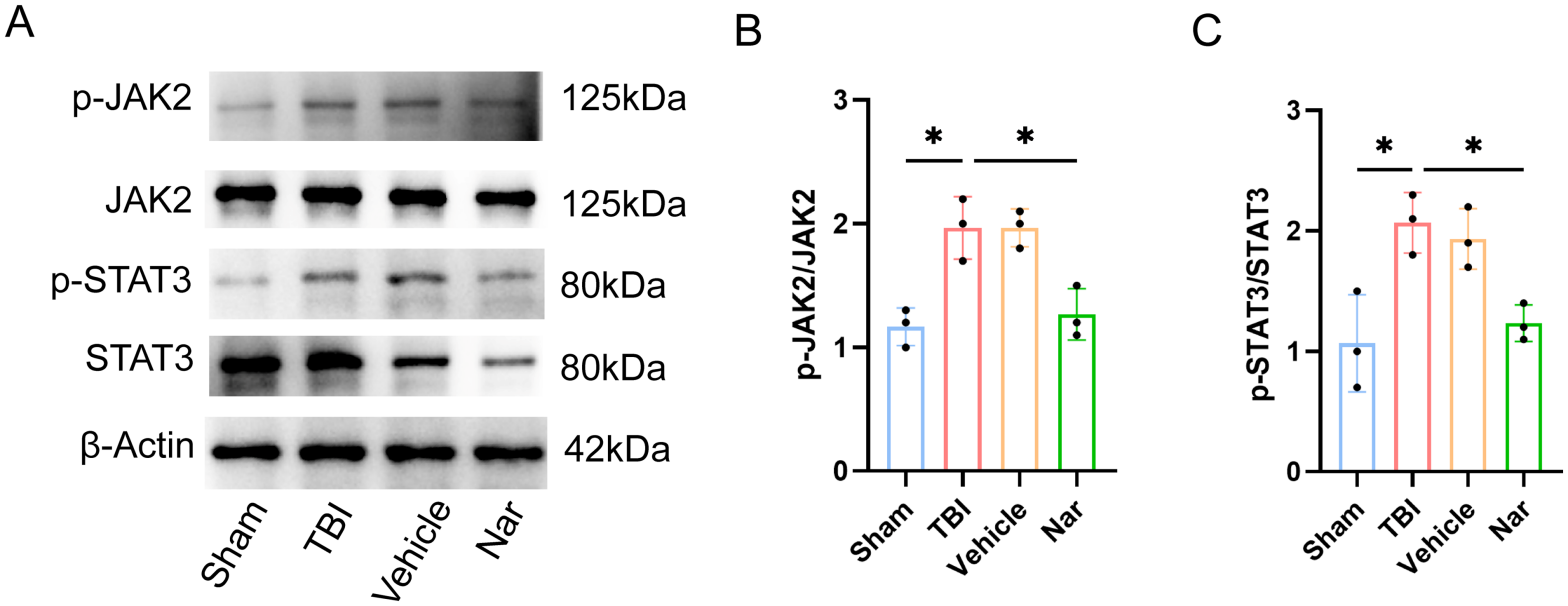
Nar inhibits JAK2/STAT3 signaling pathway activation post-TBI. **(A–C)** Western blot bands and phosphorylation ratio analysis of p-JAK2 (Tyr1007/1008), total JAK2, p-STAT3 (Tyr705), and STAT3 proteins in vivo. ns; *p* > 0.05; *p ≤ 0.05; **p ≤ 0.01; ***p ≤ 0.001; ****p ≤ 0.0001; *n* = 3.

The neuroprotective effects of Nar against TBI may be mediated through the JAK–STAT pathway. We further investigated whether Nar exerts its therapeutic actions via the JAK2-STAT3 signaling axis in TBI (Figures 6A–C). Western blot analysis provided additional mechanistic evidence: while total JAK2 and STAT3 protein levels remained consistent across sham, TBI, and Nar-treated groups, their phosphorylated forms (p-JAK2 and p-STAT3) showed significant elevation post-TBI, indicating pathway activation following injury. Crucially, Nar administration substantially reduced phosphorylation levels of these signaling molecules. These results demonstrate that Nar inhibits TBI-induced activation of the key JAK2-STAT3 signaling cascade.

## 4 Discussion

TBI represents an acute clinical condition with unacceptably high mortality and disability rates, posing a major global neuroscience challenge due to the absence of effective therapies (35, 36). In the cascade-amplifying pathological reactions following TBI, cerebral hypoxia induced by tissue hemorrhage and dysregulated neuroinflammation form a vicious cycle, directly causing irreversible neuronal damage (37, 38). This neuronal injury constitutes the core pathological basis of long-term neurological dysfunction (39). The pathophysiology of TBI involves complex interrelated mechanisms, predominantly featuring oxidative stress and neuroinflammation (40). These dual pathological drivers synergistically activate apoptotic signaling pathways, creating a microenvironment that accelerates neuronal death (41). Collectively, these factors exacerbate neuronal apoptosis, amplify primary injury severity, and significantly impede post-injury neurological recovery (42). Consequently, mitigating neuroinflammation is critical for TBI therapeutic intervention. This study provides the first evidence that Narirutin—a citrus-derived flavonoid—significantly reduces pro-inflammatory cytokine release (TNF-α, IL-1β, IL-6) and remodels the immune microenvironment in injured regions through selective inhibition of the JAK/STAT signaling axis.

Previous studies have shown that the STAT3 signaling pathway plays a key role in the inflammatory response after TBI (43). In neuroinflammation, certain pro-inflammatory cytokines, such as IL-6, IL-1β, and TNF-α, can activate JAK family kinases in microglia, particularly JAK2. Due to JAK activation, specific tyrosine residues on cytokine receptors are phosphorylated (44). Then, these phosphorylated receptors recruit and activate STAT proteins, mainly STAT3. Once activated, STAT proteins translocate to the nucleus and control the expression of target genes involved in the inflammatory response. When STAT3 phosphorylation is significantly inhibited, the transcriptional control of pro-inflammatory proteins is weakened, neuroinflammation is alleviated, and glial cell activation is reduced. During neuroinflammation, the JAK/STAT pathway in microglia has both beneficial and harmful effects (45). On the one hand, the activation of STAT3 in microglia can stimulate the production of anti-inflammatory molecules and neuroprotective factors, which is conducive to tissue repair and the resolution of inflammation. On the other hand, the continuous or excessive activation of the JAK/STAT pathway in microglia can lead to the release of neurotoxic factors and the persistence of inflammation, ultimately resulting in neuronal damage (46).

As classical resident immune cells within the CNS, microglia constitute essential components for maintaining CNS homeostasis and play a pivotal role in TBI-induced neuroinflammation (47, 48). Upon activation, microglia participate in post-TBI inflammatory responses through the release of pro-inflammatory cytokines including IL-1 and TNF (49). It is well established that microglia polarize into two distinct phenotypes: the pro-inflammatory M1-type and the anti-inflammatory M2-type (50, 51). M1 microglia secrete pro-inflammatory mediators (e.g., TNF-α, IL-6) to combat pathogens, yet their persistent activation can exacerbate tissue damage (45). Conversely, M2 microglia release immunomodulatory cytokines including IL-4 and IL-10, which suppress excessive production of pro-inflammatory factors (such as IL-1 and TNF) and maintain homeostasis during neuroinflammation (52). Consequently, modulating the balance between M1 and M2 polarization represents a critical therapeutic strategy for mitigating neuroinflammatory pathology (53, 54).

Research demonstrates that Nar possesses potent anti-inflammatory and antioxidant properties, effectively mitigating inflammation in diverse neurological disorders including ischemic stroke, Parkinson’s disease, and depression (23, 27, 30, 55). Moreover, Nar exhibits significant anti-inflammatory efficacy in non-neurological conditions such as colitis and allergic rhinitis (56, 57). Recent studies indicate that Nar exerts anti-inflammatory effects through suppression of the NLRP3 inflammasome (58). Furthermore, Nar mediates anti-inflammatory actions via the PI3K/AKT signaling pathway (59).

It is important to acknowledge the limitations of this study. Firstly, we created a model of only moderate TBI and used it to investigate the anti-neuroinflammatory effect of Nar. It is necessary to establish other TBI models of different severities (milder and more severe) for future investigations. Thirdly, we only evaluated some protein factors in the JAK, STAT and apoptosis-related pathways. The impact of Nar on other signaling pathways requires further experimental exploration, and more detailed analysis will be valuable in the future. More importantly, our study mainly focused on the acute phase of inflammation after TBI. Whether Nar can produce a lasting neuroprotective effect remains to be further verified. Additionally, this study has not been validated in human tissues and no clinical trials have been conducted to assess the therapeutic effect of Nar. Therefore, future research should further explore the role of Nar in the chronic phase of TBI and evaluate the feasibility of its clinical application.

In our study, Nar treatment significantly reduced neuronal apoptosis and markedly improved previously impaired neurological function. These findings indicate that Nar not only functions as an anti-inflammatory agent but also exhibits neurorestorative potential, ameliorating TBI-induced neurological deficits. *In vivo* experiments revealed a substantial increase in Iba1-positive cells within the hippocampal region of TBI model mice. Notably, Nar reversed microglial proliferation in the hippocampus. These results demonstrate that Nar suppresses the proportion of M1-polarized microglia in post-TBI brains. Furthermore, we observed Nar-mediated inhibition of TNF and IL-6 expression in microglia—consistent with established research linking M1 activation to pro-inflammatory cytokine release (60).

## Data Availability

The raw data supporting the conclusions of this article will be made available by the authors, without undue reservation.
